# Efficacy of cefiderocol in combination with xeruborbactam versus taniborbactam against cefiderocol-resistant NDM-producing *Pseudomonas aeruginosa*

**DOI:** 10.1128/aac.00857-25

**Published:** 2025-10-10

**Authors:** Kaan Kocer, Truong Nhat My, Bui Tien Sy, Lisa Göpel, Thirumalaisamy P. Velavan, Le Huu Song, Silke Peter, Sébastien Boutin, Adesola Olalekan, Dennis Nurjadi

**Affiliations:** 1 Institute of Medical Microbiology, University of Lübeck and University Hospital Schleswig-Holstein Campus Lübeck, Lübeck, Germany; 2 German Center for Infection Research (DZIF), Partner Site Hamburg-Lübeck-Borstel-Riems, Lübeck, Germany; 3 Vietnamese-German Centre for Medical Research (VG-CARE), Hanoi, Vietnam; 4 108 Military Central Hospital, Hanoi, Vietnam; 5 Faculty of Medicine, Duy Tan University 374802, Da Nang, Vietnam; 6 Institute of Tropical Medicine, Universitätsklinikum Tübingen 27203, Tübingen, Germany; 7 German Center for Infection Research (DZIF), Partner Site Tübingen, Tübingen, Germany; 8 Institute of Medical Microbiology and Hygiene, Universitätsklinikum Tübingen 27203, Tübingen, Germany; 9 Airway Research Center North (ARCN), German Center for Lung Research (DZL) 542891, Lübeck, Germany; 10 Department of Medical Laboratory Science, College of Medicine, University of Lagos 70670, Lagos, Nigeria; University of Fribourg, Fribourg, Switzerland

**Keywords:** *Pseudomonas aeruginosa*, NDM, taniborbactam, xeruborbactam, cefiderocol

## Abstract

We evaluated the *in vitro* activity of cefiderocol in combination with either taniborbactam or xeruborbactam against cefiderocol-resistant, *bla*_NDM_-positive *Pseudomonas aeruginosa* clinical isolates from Vietnam and Nigeria. Taniborbactam restored cefiderocol susceptibility in most isolates (20/21, 95.2%), while xeruborbactam had no effect. Resistance was reversed by dipicolinic acid, which confirmed New-Delhi Metallo-β-Lactamase (NDM-mediated) resistance. One isolate that was unresponsive to taniborbactam carried multiple copies of *bla*_NDM-1_. These findings highlight the species-specific limitations of xeruborbactam in *P. aeruginosa*.

## INTRODUCTION

Metallo-beta-lactamase (MBL)-producing gram-negative bacteria, particularly those expressing NDM, present a major clinical challenge due to the limited availability of alternative treatment options. One potential treatment option is cefiderocol, a siderophore-conjugated cephalosporin antibiotic with stability against hydrolysis by MBL. However, NDM production in Gram-negative bacteria is associated with elevated cefiderocol minimum inhibitory concentrations (MICs), and cefiderocol non-susceptibility is particularly high in NDM-producing *Pseudomonas aeruginosa* (1). In high-income countries, carbapenem resistance in *P. aeruginosa* is typically driven by mutations that lead to the overproduction of efflux pumps and the loss of porin rather than NDM production (2). In contrast, NDM- or other carbapenemase-producing *P. aeruginosa* is frequently encountered in many low- and middle-income countries (3). In low-income countries, the lack of alternatives to treat these extensively drug-resistant *P. aeruginosa* has led to the extensive use of colistin. However, the toxicity of colistin is a major issue that could be resolved by novel substances that can overcome the underlying resistance mechanisms, such as MBL production.

Currently, licensed novel β-lactamase inhibitors, e.g., avibactam, relebactam, and vaborbactam, lack activity against MBLs. However, two novel boronate-based β-lactamase inhibitors, taniborbactam and xeruborbactam, have shown promise in inhibiting MBLs, with the exception of NDM-9 and NDM-30 variants and IMP for taniborbactam and some IMP variants for xeruborbactam (4–6). While existing data on the *in vitro* activity of xeruborbactam in combination with β-lactams primarily focuses on the meropenem-xeruborbactam combination (7–9), phase 1 clinical trials evaluating xeruborbactam in combination with ceftibuten and cefiderocol are currently underway (NCT06079775 and NCT06547554, respectively). Preliminary results suggest that when combined with cefiderocol, xeruborbactam reduces the MIC of cefiderocol in Enterobacterales and *Acinetobacter baumannii* (10, 11). However, Le Terrier *et al*. showed that, when combined with cefepime, xeruborbactam was less effective than taniborbactam in reducing the MIC values of β-lactams in MBL-producing *P. aeruginosa* recombinant strains (12).

The efficacy of combining taniborbactam or xeruborbactam with cefiderocol against NDM-producing *P. aeruginosa* clinical isolates remains unexplored. This study investigates the *in vitro* activity of cefiderocol in combination with taniborbactam or xeruborbactam against NDM-producing, cefiderocol-resistant *P. aeruginosa* isolates obtained from clinical specimens in Vietnam and Nigeria.

34 NDM-producing *P. aeruginosa* isolates from Nigeria were obtained as part of a previous study (13). In Vietnam, 12 NDM-producing *P. aeruginosa* isolates were obtained from an unpublished collection of carbapenem-resistant isolates recovered between January and December 2021 from the intensive care units at the 108 Military Central Hospital in Hanoi. Detection of *P. aeruginosa* was performed in the routine microbiological diagnostic laboratory in Vietnam, using MALDI-TOF MS for identification and VITEK 2 for antimicrobial susceptibility testing. The isolates collected in Vietnam were sequenced using a hybrid methodology, and draft genomes and annotations were performed as previously published (see Supplementary Methods for more details) (14). To minimize sampling bias, only one isolate per patient was included from both study sites.

Cefiderocol susceptibility testing was performed on all isolates using the broth microdilution (BMD) method with iron-depleted cation-adjusted Mueller-Hinton broth (ID-CA-MHB), prepared in-house following a previously published protocol (15). For isolates resistant to cefiderocol, additional BMD testing was performed to evaluate the activity of cefiderocol in combination with a fixed concentration (4 µg/mL) of either xeruborbactam (Xeruborbactam disodium, MedChemExpress, USA) or taniborbactam (Taniborbactam hydrochloride, Biozol, Germany). To further assess the role of NDM in cefiderocol resistance, BMD was performed also using ID-CA-MHB supplemented with 100 mg/L dipicolinic acid (DPA), a known inhibitor of MBL activity. The EUCAST version 15.0 clinical breakpoint for cefiderocol against *P. aeruginosa* (>2 mg/L) was applied to interpret the results of both combinations. The reference strains *P. aeruginosa* ATCC27853 were used as a control for all susceptibility testing. Additionally, *E. coli* NCTC 13353, *K. pneumoniae* ATCC BAA-2814, and two previously characterized cefiderocol-resistant non-*Pseudomonas* isolates -*bla*_NDM-5_-positive *Enterobacter cloacae* (16) and *bla*_NDM-1_-positive *Klebsiella pneumoniae*- from our earlier studies and an unpublished cohort were included as external comparators to confirm the activity of xeruborbactam and taniborbactam in our BMD (Table 1).

**TABLE 1:** Susceptibility testing results of non-*Pseudomonas* quality control strains^*a*^

Non-*Pseudomonas* control strains	CFD	CFD + XER	CFD + TAN	CFD + DPA	CEP	CEP + TAN	MEM	MEM + XER	MLST
*E. coli* NCTC13353	0.25	–^*b*^	<=0.03	–	>=64	0.5	–	–	–
*K. pneumoniae* BAA-2814	1	0.125	–	–	–	–	>=64	0.06	–
*K. pneumoniae* Klpn003	8	2	2	1	–	–	–	–	101
*E. cloacae* etcl_1	4	1	0.5	0.25	–	–	–	–	96

^*a*^ CFD, cefiderocol; XER, xeruborbactam; TAN, taniborbactam; DPA, dipicolinic acid; CEP, cefepime; MEM, meropenem.

^*b*^ –, isolates do not belong to any particular cluster (singleton).

The study included a total of 46 NDM-positive isolates. Of these, 21 isolates (45.7%) were resistant to cefiderocol (Table 2), with MIC values ranging from 4 to 32 mg/L. These results were consistent with previous studies reporting high cefiderocol resistance rates in NDM-positive *P. aeruginosa* (17). All isolates harbored the *bla*_NDM-1_ gene. Detailed genotypic resistance data for all isolates can be found in the Data S1.

**TABLE 2:** Susceptibility testing of cefiderocol and its combinations with either xeruborbactam (XER), taniborbactam (TAN), or DPA, for the studied NDM-positive *Pseudomonas aeruginosa* isolates and NDM-positive non-*Pseudomonas* quality control strains

*P. aeruginosa* isolates	MIC (mg/L)				MLST	Cluster^*a*^
	Cefiderocol	Cefiderocol +XER	Cefiderocol +TAN	Cefiderocol +DPA		
X273	32	32	16	0.5	233	C01
X351	0.5	0.5	0.06	<=0.03	1420	–^*b*^
X444	1	0.5	0.06	<=0.03	308	C04
X445	8	8	2	0.5	233	C01
X474	4	4	1	0.5	308	C03
X503	4	4	0.5	0.25	308	C03
X549	2	2	0.5	0.5	308	C04
X583	4	4	2	0.5	308	C03
X734	4	4	0.5	0.25	308	C03
X888	4	4	1	0.5	308	C03
X951	1	1	0.5	0.06	235	–
X2041	2	2	0.25	<=0.03	308	C03
X010_19	4	4	0.5	0.25	773	C02
X109_19	4	4	0.5	0.25	773	C02
X123_19	8	8	1	0.5	773	C02
X132_19	4	4	0.5	0.25	773	C02
X159_19	2	2	0.25	0.125	773	C02
X193_19	1	1	0.5	0.125	773	–
X201_19	8	8	1	0.125	773	C02
X202_19	16	16	2	0.5	773	C02
X204_19	8	8	1	0.25	773	C02
X205_19	4	4	2	1	773	C02
X230_19	4	4	0.5	0.06	773	C02
X235_19	2	2	0.5	0.25	773	–
X239_19	0.5	0.5	0.125	<=0.03	773	C02
X257_19	0.25	0.125	<=0.03	<=0.03	773	C02
X263_19	0.25	0.25	0.125	<=0.03	773	C02
X265_19	8	8	2	0.25	773	C02
X274_19	1	1	0.25	<=0.03	773	C02
X302_19	2	2	0.5	0.125	773	C02
X303_19	2	2	0.5	0.06	773	C02
X306_19	4	4	1	0.25	773	–
X309_19	4	4	0.5	0.5	773	C02
X312_19	1	1	0.5	0.125	773	C02
X314_19	1	1	0.25	0.06	773	C02
X324_19	2	2	1	<=0.03	773	–
X331_19	2	2	0.5	0.125	773	C02
X332_19	0.06	0.06	<=0.03	<=0.03	773	C02
X338_19	8	8	0.5	0.5	773	–
X341_19	1	1	0.5	<=0.03	773	–
X344_19	2	2	0.25	<=0.03	773	–
X352_19	2	2	0.5	0.06	773	C02
X359_19	4	4	0.5	0.5	773	C02
X361_19	1	1	0.25	<=0.03	773	C02
X362_19	0.5	0.5	0.125	<=0.03	773	C02
X363_19	0.5	0.5	0.125	<=0.03	773	C02

^*a*^ The cluster is based on average nucleotide identity (ANI) of 99.99%.

^*b*^ –, isolates do not belong to any particular cluster (singleton).

The isolates belonged to five different sequence types (STs), with ST773 being the most prevalent overall. All of the isolates from Nigeria (34/34, 100%) belonged to ST773, while the most prevalent ST in Vietnam was ST308 (8/12, 66.6%) (Fig. S1). Cefiderocol-resistant isolates were identified in three different STs: ST773, ST308, and ST233. Average nucleotide identity analysis using a cut-off value of 99.99% revealed four genetic clusters, with cefiderocol-resistant isolates present in three different clusters, and two resistant isolates not belonging to any cluster. These findings suggest that cefiderocol resistance occurs in diverse genetic clones and is not limited to a single clonal lineage.

As NDM activity has previously been attributed to cefiderocol resistance (18), we investigated whether the inhibition of NDM by DPA could restore the cefiderocol susceptibility. Indeed, in the presence of DPA, the cefiderocol MICs for all resistant isolates were reduced to susceptible levels (Table 2), indicating that NDM was an important contributor to resistance.

We also investigated previously described cefiderocol resistance determinants beyond NDM (19). Comparative SNP analysis of cefiderocol-resistant versus susceptible isolates did not reveal unique mutations in most resistant isolates. However, some resistant isolates carried mutations previously associated with cefiderocol resistance (Data S3), suggesting that, while NDM was likely the primary resistance mechanism, other mechanisms may also contribute to the overall resistance phenotype. Two isolates (X273 and X445) carried the highest number of mutations and had elevated cefiderocol MICs (32 mg/L and 8 mg/L, respectively). However, in both cases, the MICs decreased to 0.5 mg/L in the presence of DPA. The full data set of mutations present only in the resistant isolates is available in the Data S3.

Next, we evaluated whether adding taniborbactam or xeruborbactam could restore cefiderocol activity. Except for one isolate (X273), which had the highest cefiderocol MIC in the cohort, the addition of taniborbactam substantially reduced cefiderocol MICs, shifting them into the susceptible range based on EUCAST breakpoints. In contrast, xeruborbactam had no impact on cefiderocol MICs in NDM-producing *P. aeruginosa* (Table 2). This species-specific lack of efficacy in *P. aeruginosa* aligns with findings by Le Terrier *et al*., who reported reduced xeruborbactam activity in recombinant *P. aeruginosa* strains, potentially due to active efflux via the wild-type MexAB–OprM multidrug efflux system (12). In our cohort, three types of frameshift mutations in *mexR* and *nalD*, the regulators of MexAB–OprM, were identified in 11 isolates with varying cefiderocol susceptibility (Data S2). However, xeruborbactam did not significantly change the cefiderocol MICs of these 11 isolates, indicating that these mutations have no impact on xeruborbactam activity.

We further investigated the potential underlying resistance mechanism in the isolate X273, in which the taniborbactam could not revert the cefiderocol MIC to susceptible levels. WGS analysis revealed multiple copies of the *bla*_NDM-1_ gene. Previous reports have linked high cefiderocol MICs to increased *bla*_NDM_ gene copy number (18), suggesting that a similar mechanism could explain the persistent resistance despite the addition of taniborbactam. To support this hypothesis, we quantified *bla*_NDM-1_ mRNA expression in X273 and its genetically closest strain in the cohort, X445, as previously described (20). The expression level in X273 was 3- to 4-fold higher than in X445, suggesting that the higher NDM expression is responsible for the elevated cefiderocol MIC. Furthermore, testing with higher concentrations of taniborbactam reduced the cefiderocol MIC of X273 further: to 2 mg/L at 8 mg/L taniborbactam and to 1 mg/L at 16 mg/L taniborbactam.

One limitation of our study is the low genetic diversity of the isolates, which may restrict how widely the findings can be applied. However, we focused on *bla*_NDM_-positive *P. aeruginosa*, which restricts the global population. Our findings demonstrate that cefiderocol resistance in *bla*_NDM_-positive *P. aeruginosa* is primarily driven by NDM activity and cannot be overcome by combining it with xeruborbactam. Furthermore, a similar finding has recently been reported in IMP-producing *P. aeruginosa* (21). These highlight important differences in the efficacy of novel β-lactamase inhibitors. Evaluations of new antimicrobial combinations specific to pathogens are needed, especially in the context of NDM-mediated resistance.

## SUPPLEMENTAL MATERIAL

Data S1 aac.00857-25-s0001.xlsx Antimicrobial resistance genes of 46 *P. aeruginosa* isolates and two non-Pseudomonas quality control isolates included in this study.

Data S2 aac.00857-25-s0002.xlsx SNPs found in the MexAB-OprM multidrug efflux system of each studied isolate, using PAO1 as the reference genome.

Data S3 aac.00857-25-s0003.xlsx Overview of mutations in the genes of interest identified only in cefiderocol-resistant *P. aeruginosa* isolates.

Fig. S1 aac.00857-25-s0004.docx Average nucleotide identity-based phylogenetic dendrogram of 46 blaNDM-1-positive *P. aeruginosa* isolates.

Supplemental material aac.00857-25-s0005.docx Supplemental methods.

ASM does not own the copyrights to Supplemental Material that may be linked to, or accessed through, an article. The authors have granted ASM a non-exclusive, world-wide license to publish the Supplemental Material files. Please contact the corresponding author directly for reuse.
