# Supplementary material for: Efficacy of cefiderocol in combination with xeruborbactam versus taniborbactam against cefiderocol-resistant NDM-producing Pseudomonas aeruginosa
Source: Antimicrob Agents Chemother. 2025 Oct 10;69(11):e00857-25. doi: 10.1128/aac.00857-25 (PMC12587528; doi:10.1128/aac.00857-25)
Supplement: Fig. S1 — Average nucleotide identity-based phylogenetic dendrogram of 46 blaNDM-1-positive P. aeruginosa isolates. [file aac.00857-25-s0004.docx]

**
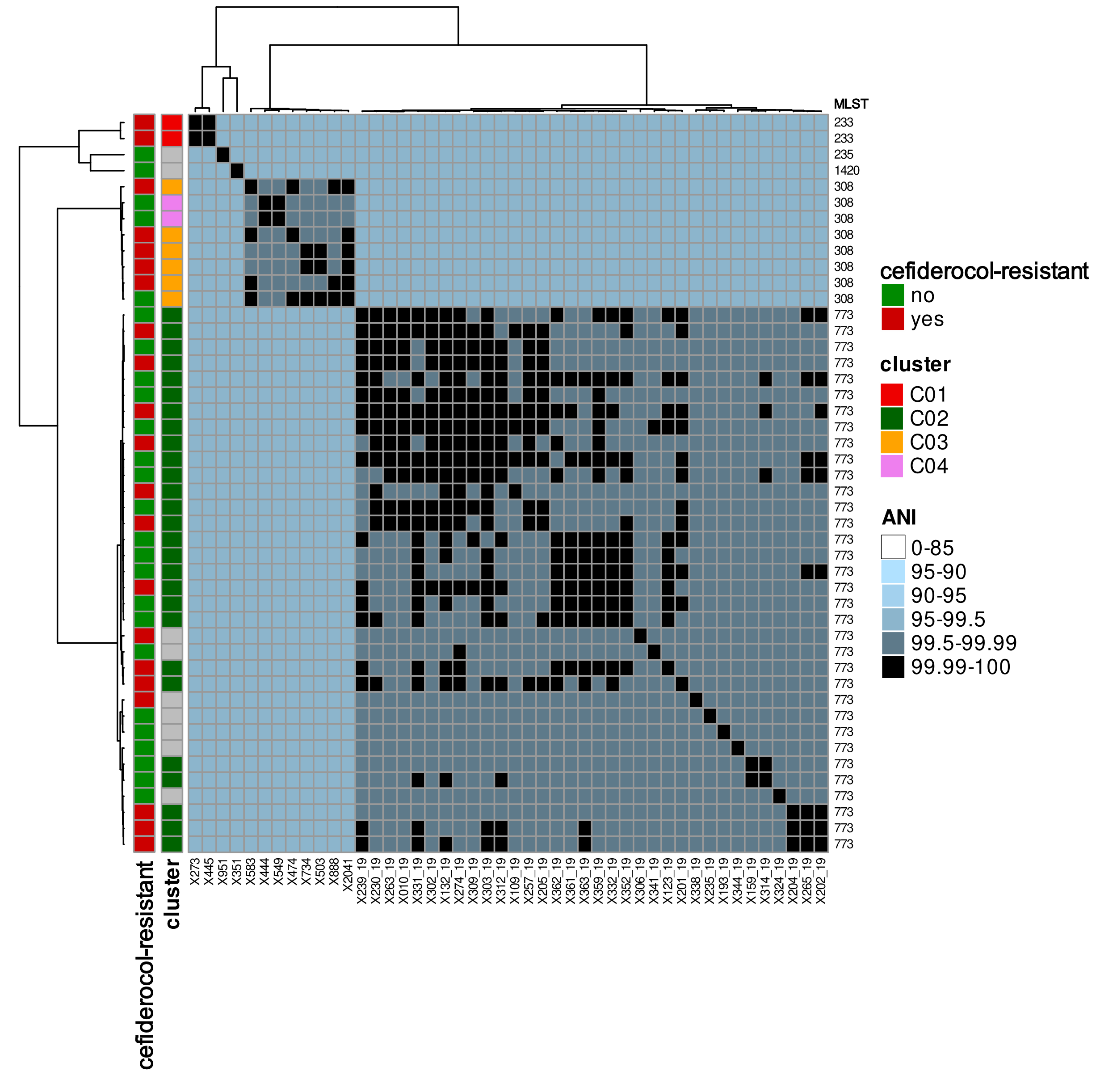
Figure S1** Average nucleotide identity (ANI)-based phylogenetic dendrogram of 46 *bla*_NDM-1-_positive *Pseudomonas aeruginosa* isolates. Whole-genome sequences were analysed using ANIclustermap (v1.1.0), with clustering based on pairwise ANI values. The coloured bar to the left of the dendrogram indicates cefiderocol susceptibility phenotype: red indicates resistance (MIC > 2 mg/L), green indicates susceptiblity (MIC ≤ 2 mg/L), based on EUCAST v15.0 breakpoints.
