# Supplementary material for: Efficacy of cefiderocol in combination with xeruborbactam versus taniborbactam against cefiderocol-resistant NDM-producing Pseudomonas aeruginosa
Source: Antimicrob Agents Chemother. 2025 Oct 10;69(11):e00857-25. doi: 10.1128/aac.00857-25 (PMC12587528; doi:10.1128/aac.00857-25)
Supplement: Supplemental material — Supplemental methods. [file aac.00857-25-s0005.docx]

**Supplementary Methods**

Library preparation was performed using Nextera DNA Flex Library Prep Kit (Illumina) and sequencing was done on a MiSeq Illumina platform (short-read sequencing, 2 ×301 bp). Post-sequencing procedure was performed as follows: Raw sequences were controlled for quality and adapter removal using fastp (v0·23·2 with parameters -q = 30 and -l = 45) (1). Clean reads were then used to create de novo assembly using SPAdes 3.15.5 (with the option —careful and—only-assembler) (2). Draft genomes were curated by removing contigs with a length <500 bp and/or coverage <10×. The quality of the final draft was quality-controlled using Quast (v5.0.2) (3).

The species identification of each draft genome was done using mash (sub-command screen) by screening each draft genome to a database composed of a representative genome of each species present in the Microbial Genomes resource (<https://www.ncbi.nlm.nih.gov/genome/microbes/>). MLST was define using the tool mlst v2.23.0 which use the database PubMLST (Seemann T, Github <https://github.com/tseemann/mlst>) (4). ANI was calculated using an all-vs-all analysis of the genomes using ANIclustermap (v.1.3.0) (5).

The complete draft genomes were processed through available databases using Abricate (minimum identity 90% and minimum coverage 80%) (<https://github.com/tseemann/abricate>) to identify antimicrobial resistance (NCBI, CARD, ARG-ANNOT, ResFinder, MEGARES databases), virulence genes (VFDB databases) and plasmid type (PlasmidFinder database) to identify the Inc type of the plasmid (6-11).

To investigate additional cefiderocol resistance mechanisms, we performed a single nucleotide polymorphism (SNP) analysis focusing on the genes previously described as relevant for cefiderocol susceptibility (12). Initial mapping was performed against the wild-type reference strain PAO1 to facilitate the annotation of the SNPs and SNP calling was performed using snippy pipeline (https://github.com/tseemann/snippy). After identifying these SNPs, we compared the SNP profiles of cefiderocol-resistant and cefiderocol-susceptible isolates within the cohort. We screened for mutations that were present exclusively in at least one of the resistant isolates, while excluding those that were also found in any of the susceptible isolates. The complete list of unique mutations observed in the genes of interest in cefiderocol-resistant isolates is provided in Supplementary Data S3.

**References**

1. Chen S, Zhou Y, Chen Y, Gu J. 2018. fastp: an ultra-fast all-in-one FASTQ preprocessor. Bioinformatics 34:i884-i890.

2. Prjibelski A, Antipov D, Meleshko D, Lapidus A, Korobeynikov A. 2020. Using SPAdes De Novo Assembler. Current Protocols in Bioinformatics 70:e102.

3. Gurevich A, Saveliev V, Vyahhi N, Tesler G. 2013. QUAST: quality assessment tool for genome assemblies. Bioinformatics 29:1072-1075.

4. Jolley KA, Maiden MCJ. 2010. BIGSdb: Scalable analysis of bacterial genome variation at the population level. BMC Bioinformatics 11:595.

5. Shimoyama Y. 2022. ANIclustermap: A tool for drawing ANI clustermap between all-vs-all microbial genomes.

6. Feldgarden M, Brover V, Haft DH, Prasad AB, Slotta DJ, Tolstoy I, Tyson GH, Zhao S, Hsu CH, McDermott PF, Tadesse DA, Morales C, Simmons M, Tillman G, Wasilenko J, Folster JP, Klimke W. 2019. Validating the AMRFinder Tool and Resistance Gene Database by Using Antimicrobial Resistance Genotype-Phenotype Correlations in a Collection of Isolates. Antimicrob Agents Chemother 63.

7. Jia B, Raphenya AR, Alcock B, Waglechner N, Guo P, Tsang KK, Lago BA, Dave BM, Pereira S, Sharma AN, Doshi S, Courtot M, Lo R, Williams LE, Frye JG, Elsayegh T, Sardar D, Westman EL, Pawlowski AC, Johnson TA, Brinkman FSL, Wright GD, McArthur AG. 2016. CARD 2017: expansion and model-centric curation of the comprehensive antibiotic resistance database. Nucleic Acids Research 45:D566-D573.

8. Zankari E, Hasman H, Cosentino S, Vestergaard M, Rasmussen S, Lund O, Aarestrup FM, Larsen MV. 2012. Identification of acquired antimicrobial resistance genes. J Antimicrob Chemother 67:2640-4.

9. Gupta SK, Padmanabhan BR, Diene SM, Lopez-Rojas R, Kempf M, Landraud L, Rolain JM. 2014. ARG-ANNOT, a new bioinformatic tool to discover antibiotic resistance genes in bacterial genomes. Antimicrob Agents Chemother 58:212-20.

10. Chen L, Zheng D, Liu B, Yang J, Jin Q. 2016. VFDB 2016: hierarchical and refined dataset for big data analysis--10 years on. Nucleic Acids Res 44:D694-7.

11. Carattoli A, Zankari E, García-Fernández A, Voldby Larsen M, Lund O, Villa L, Møller Aarestrup F, Hasman H. 2014. In silico detection and typing of plasmids using PlasmidFinder and plasmid multilocus sequence typing. Antimicrob Agents Chemother 58:3895-903.

12. Sastre-Femenia M, Fernández-Muñoz A, Gomis-Font MA, Taltavull B, López-Causapé C, Arca-Suárez J, Martínez-Martínez L, Cantón R, Larrosa N, Oteo-Iglesias J, Oliver A. 2025. Spanish nationwide survey of Pseudomonas aeruginosa cefiderocol susceptibility and resistance mechanisms. Int J Antimicrob Agents 66:107563.
